# Depression and anxiety prevalence in nursing staff during the COVID-19 pandemic

**DOI:** 10.1097/01.NUMA.0000752784.86469.b9

**Published:** 2021-05-28

**Authors:** Jan Serrano, Sameer Hassamal, Sunita Hassamal, Fanglong Dong, Michael Neeki

**Affiliations:** At Arrowhead Regional Medical Center in Colton, Calif., **Jan Serrano** is a nurse scientist, **Sameer Hassamal** is a consultation and liaison psychiatrist, **Sunita Hassamal** is internal medicine residency faculty, and **Michael Neeki** is core faculty and director of research. At Western University of Health Sciences in Pomona, Calif., **Fanglong Dong** is an associate professor.

## Abstract

This article reviews a study to determine the effects of COVID-19 on the rate of depression and anxiety in nurses and to identify potential strategies that nurse leaders can implement to reduce depression and anxiety in their staff.

**Figure:**
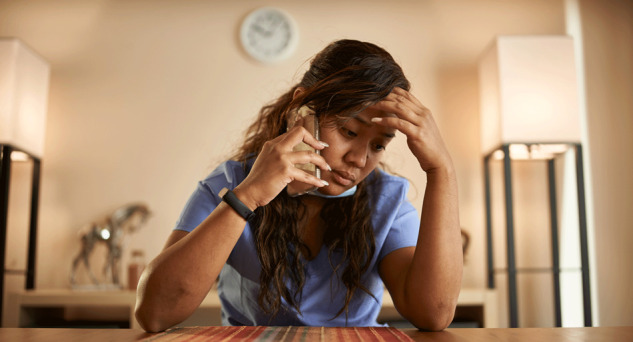
No caption available.

The World Health Organization declared COVID-19 a pandemic on March 11, 2020, as the novel virus spread rapidly across the globe.^1^ As the number of COVID-19 cases grew, the US experienced a parallel increase in mental health crisis, with rates of depression and anxiety tripling.^2^ Recent studies suggest that healthcare workers are at a higher risk for experiencing depression and anxiety.^2^ Currently, nurses make up more than half of the world's healthcare workers.^1^ Nurses are at the front lines providing care and support, yet are vulnerable to fear, depression, and anxiety.^3^

Increased rates of depression and anxiety among nurses working in the COVID-19 environment isn't unexpected because the clinical trajectory of COVID-19 still isn't well understood and the risks are unclear. Psychological stressors contributing to mental health distress have ranged extensively during the pandemic, including shortages of personal protective equipment (PPE), limited access to COVID-19 testing supplies, fear of disease transmission at work, fear of lack of employer support if COVID-19 is contracted, depression when patients succumb to the virus, ethical decisions regarding care rationing, and drastic changes in workplace practice.^3^,^4^ Additionally, nurses chose to limit fluid intake to reduce toileting needs and worked longer periods of time in an exposed area to reduce the number of PPE changes. This group has also assumed added responsibilities, performing ancillary roles to minimize staff exposures.^5^ Persistent exposure increases the risk of fatigue and anxiety.^5^ Furthermore, the frequency and duration of nurses' exposure to COVID-19 is correlated with their fear level.^5^

In addition, organizational policies have frequently changed as new knowledge of disease transmission and treatment options emerge. Frequent policy changes on prevention strategies and treatment plans, and poor communication of changes or accessibility of information, negatively affect nurses' mental health.^3^ Moreover, this group has also had to deal with social isolation and ostracization by family, friends, and the public due to fear of exposure and transmission of COVID-19.^4^,^6^-^8^ All of these factors place a further psychological burden on nurses.^9^,^10^ An editorial from China reported increased nurse suicides resulting from the psychological pressure of caring for COVID-19 patients.^4^

The aims of this study were to determine the effects of COVID-19 on the prevalence of depression and anxiety in nurses and to identify potential strategies that nurse leaders can implement to reduce depression and anxiety in their staff.

## Methods

This study is part of a larger study that was conducted at Arrowhead Regional Medical Center (ARMC) to determine the prevalence of depression and anxiety and elucidate associations between sociodemographic variables, depression, and anxiety. ARMC is a 456-bed university-affiliated teaching public hospital that serves as a safety net hospital for the uninsured and underserved population in San Bernardino County, Calif.^11^

The design for the original study was an anonymous cross-sectional convenience sample survey using an online survey platform. The survey's web link was emailed to nursing administration clinical assistants (CAs), student nurses (SNs), licensed vocational nurses (LVNs), registered nurses (RNs), and advanced practice nurses (APNs). Prospective participants were recruited from an all-users email sent by ARMC's chief executive officer. The survey was open for responses between April 30 and May 22, 2020. Inclusion criteria included being older than age 18; working in the hospital as a CA, SN, LVN, RN, or APN; being willing and able to give informed consent; and being able to complete the survey in English. This study was approved by ARMC's Institutional Review Board. During the study period, the number of confirmed COVID-19 cases doubled from 2,058 to 4,146 in San Bernardino County.

The Patient Health Questionnaire 9 (PHQ-9) was used to measure self-reported levels of depression. The PHQ-9 consists of nine questions to measure the frequency of depressive symptoms over the past 2 weeks on a four-point Likert scale ranging from 0 (not at all) to 3 (nearly every day). Scores are interpreted as 0 to 4, normal; 5 to 9, mild; 10 to 14, moderate; 15 to 19, moderately severe; and 20 to 27, severe. PHQ-9 scores were categorized into two groups: <10 and ≥10. A PHQ-9 score ≥10 is categorized as major depression and is 88% sensitive and specific.^12^

The General Anxiety Disorder 7 (GAD-7) scales were used to measure self-reported levels of anxiety. The GAD-7 consists of a seven-item questionnaire to measure the level of anxiety symptoms over the past 2 weeks using a four-point Likert scale ranging from 0 (not at all) to 3 (nearly every day). The scores are interpreted as 0 to 4, normal; 5 to 9, mild; 10 to 14, moderate; and 15 to 21, severe. GAD-7 scores were divided into two groups: <8 and ≥8.^13^ The GAD-7 is a validated tool used to diagnose generalized anxiety disorder.^10^ A GAD-7 score of ≥8 is 77% sensitive and 82% specific for a diagnosis of panic disorder, social phobia, and posttraumatic stress disorder (PTSD).^13^

Data collected from staff included sex, age, occupation, years in current position, contact with a patient suspected of having or confirmed as having COVID-19, and feeling overwhelmed by COVID-19. Participants were classified into four groups: CA/SNs, LVNs, RNs, and APNs. These groups were further divided in two categories: staff providing direct care for suspected or confirmed COVID-19 patients and staff not exposed or not providing direct care for suspected or confirmed COVID-19 patients. Questions included, “Have you been in contact with a patient either suspected to have COVID-19 or confirmed to have COVID-19?” Staff members who answered “yes” were classified as providing direct care for suspected or confirmed COVID-19 patients and those who answered “no” were classified as not providing direct care for suspected or confirmed COVID-19 patients. The question “Have you been overwhelmed by the stress of the COVID-19 pandemic?” was asked to determine perceived stress measurement. Studies have validated that perceptions of stress can be measured by asking individuals how overwhelmed they are by a situation.^14^ The PHQ-9 and GAD-7 measurements were used to measure depression and anxiety symptomatology.

All statistical analyses were conducted using statistical software.^15^ Descriptive statistics were presented as means and standard deviations for continuous variables, and frequencies and proportions for categorical variables. Chi-square statistics were conducted comparing whether nurses were overwhelmed by the stress of COVID-19 between various sociodemographic factors and scores on the PHQ-9 and GAD-7. Logistic regression analyses were conducted to examine predictors for a PHQ-9 score ≥10 and a GAD-7 score ≥8. These predictors included occupation, age, sex, years in current position, being overwhelmed by COVID-19, and being in contact with a patient either suspected to have COVID-19 or confirmed to have COVID-19. All statistical analyses were two-sided. The *P* value of ≤.05 was considered statistically significant.

## Results

A total of 472 nurse participants responded to the survey and were included in the final analysis (survey response rate = 29.5%). The majority of the participants were female (88.6%, n = 418) and the average age was 42.9 (SD = 11.6). More than two-thirds of participants were RNs or APNs (68.6%, n = 324). Overall, 48.7% (n = 227) of participants were overwhelmed by COVID-19. (See Table 1.)

**Table 1:** Demographic summary

Variable	n	%
Sex		
Female	418	88.6
Male	54	11.4
Occupation		
CA/SN and LVN	148	31.4
RN and APN	324	68.6
Overwhelmed by COVID-19?		
No	239	51.3
Yes	227	48.7
Missing response = 6		
In contact with a patient either suspected to have COVID-19 (COVID rule out) or confirmed to have COVID-19?		
No	196	42.2
Yes	269	57.9
Missing response = 7		
PHQ-9 score		
<10	366	81.0
≥10	86	19.0
Missing response = 20		
GAD-7 score		
<8	309	68.4
≥8	143	31.6
Missing response = 20		
Age		
20-39	189	41.6
40-55	184	40.5
56-75	81	17.8
Missing response = 18		
Years in current position		
0-5	203	43.3
6-9	75	16.0
10+	191	40.7
Missing response = 3		

An analysis was conducted to compare those who were stressed by COVID-19 and those who weren't stressed. LVNs, CAs, and SNs had a statistically significant higher percentage (60.3%) of self-reported stress due to COVID-19 than RNs or APNs (43.4%, *P* = .0007). Participants who didn't have contact with suspected or confirmed COVID-19 patients (*P* = .0344) had higher PHQ-9 scores (≥10, *P* = <.0001) and higher GAD-7 scores (≥8, *P* = .0001), which was associated with a higher chance of being stressed by COVID-19. (See Table 2.)

**Table 2:** Analysis of factors associated with being overwhelmed by COVID-19

	Overwhelmed by COVID-19		
Variable	No	Yes	*P* value
Occupation			.0007
CA/SN and LVN	58 (39.7%)	88 (60.3%)	
RN and APN	181 (56.6%)	139 (43.4%)	
Sex			.1245
Female	206 (50%)	206 (50%)	
Male	33 (61.1%)	21 (38.9%)	
Missing response = 6			
In contact with a patient either suspected to have COVID-19 (COVID rule out) or confirmed to have COVID-19?			.0344
Yes	112 (57.1%)	84 (42.9%)	
No	127 (47.2%)	142 (52.8%)	
Missing response = 7			
PHQ-9 score			<.0001
<10	212 (57.9%)	154 (42.1%)	
≥10	19 (22.1%)	67 (77.9%)	
Missing response = 20			
GAD-7 score			<.0001
<8	202 (65.4%)	107 (34.6%)	
≥8	29 (20.3%)	114 (79.7%)	
Missing response = 20			
Age			.5909
20-39	93 (49.5%)	95 (50.5%)	
40-55	92 (50.8%)	89 (49.2%)	
56-75	45 (56.3%)	35 (43.8%)	
Missing response = 23			
Years in current position			.9178
0-5	102 (50.3%)	101 (49.8%)	
6-9	39 (52.7%)	35 (47.3%)	
10+	98 (51.9%)	91 (48.2%)	
Missing response = 205			

The first logistic analysis was to examine predictors for participants who had a PHQ-9 score of ≥10. Those who self-reported that they were overwhelmed by COVID-19 were associated with 4.06 times increased odds (95% CI = [2.56, 6.56]) of having moderate-to-severe depression. (See Table 3.)

**Table 3:** Odds ratio and corresponding 95% confidence interval for predicting PHQ-9 ≥10

	Unadjusted		Adjusted	
Variable	OR with 95% CI	*P* value	OR with 95% CI	*P* value
CA/SN and LVN	1.83 (1.12,2.96)	.0154	1.42 (.82,2.46)	.2123
RN and APN	Reference		Reference	
Age 20-39	1.41 (.72,2.92)	.3181	.95 (.41,2.25)	.9025
Age 40-55	.99 (.49,2.08)	.9739	.84 (.4,1.84)	.652
Age 56-75	Reference		Reference	
Female vs. male	1.04 (.52,2.27)	.919	.86 (.4,1.98)	.7119
In current position 0-5 years	1.22 (.72,2.08)	.4604	1.31 (.66,2.6)	.4434
In current position 6-9 years	1.78 (.9,3.43)	.0945	2.05 (.94,4.42)	.0714
In current position 10+ years	Reference		Reference	
Overwhelmed by COVID-19? Yes vs. no	4.85 (2.85,8.62)	<.0001	4.6 (2.63,8.41)	<.0001
In contact with a patient either suspected to have COVID-19 (COVID rule out) or confirmed to have COVID-19? Yes vs. no	1.61 (.99,2.67)	.0562	1.36 (.79,2.36)	.2672

OR = odds ratio; CI = confidence interval

The second logistic analysis was to examine predictors for participants who had a GAD-7 score of ≥8. LVNs, CAs, and SNs were associated with a 1.68 times higher rate of anxiety (95% CI = [1.02, 2.76]) compared with RNs and APNs. Additionally, those who self-reported that they were overwhelmed by COVID-19 were associated with 7.31 times higher rates (95% CI = [4.53, 12.13]) of moderate-to-severe anxiety. (See Table 4.) A statistically significant difference in occupation regarding anxiety was identified: CAs/SNs, 41.4% GAD-7 ≥8; LVNs, 45%; RNs, 27.1%; and APNs, 11.1%. (See Table 5.)

**Table 4:** Odds ratio and corresponding 95% confidence interval for predicting GAD-7 ≥8

	Unadjusted		Adjusted	
Variable	OR with 95% CI	*P* value	OR with 95% CI	*P* value
CA/SN and LVN	2.03 (1.34,3.07)	.0009	1.68 (1.02,2.76)	.042
RN and APN	Reference		Reference	
Age 20-39	1.58 (.89,2.87)	.1222	1.48 (.7,3.2)	.3074
Age 40-55	1.14 (.64,2.1)	.6649	1.11 (.57,2.2)	.7548
Age 56-75	Reference		Reference	
Female vs. male	1.11 (.61,2.12)	.734	0.93 (.46,1.95)	.8524
In current position 0-5 years	1.01 (.66,1.56)	.9504	0.79 (.44,1.43)	.4402
In current position 6-9 years	1 (.54,1.79)	.9886	0.85 (.41,1.73)	.649
In current position 10+ years	Reference		Reference	
Overwhelmed by COVID-19? Yes vs. no	7.42 (4.69,12.05)	<.0001	7.31 (4.53,12.13)	<.0001
In contact with a patient either suspected to have COVID-19 (COVID rule out) or confirmed to have COVID-19? Yes vs. no	1.57 (1.04,2.38)	.0305	1.37 (.85,2.22)	.194

OR = odds ratio; CI = confidence interval

**Table 5:** Occupations

	GAD-7 score <8	GAD-7 score ≥8	*P* value = .0060
CA/SN	61 (58.7%)	43 (41.4%)	
LVN	22 (55%)	18 (45%)	
RN	218 (72.9%)	81 (27.1%)	
APN	8 (88.9%)	1 (11.1%)	

Frequency missing = 20

## Discussion

Before the COVID-19 pandemic, data regarding the effect of unprecedented high-pressure work environments on nurses' mental health were limited.^15^ In this study, nurses reported heightened stress during the COVID-19 outbreak, resulting in substantially higher levels of depression and anxiety. Similar results were noted in a study by Mo and colleagues, which identified fear as the main cause of anxiety.^5^

During the pandemic, nurses have worked under intense pressure, which can negatively affect their resilience.^16^ Long-term exposure to stressful conditions weakens nurses' resilience, resulting in an increase in anxiety levels.^5^ In this study, 79.7% of nurses reported feeling anxious. The results of reported dysfunctional levels of anxiety were comparable with a study by Labrague and De Los Santos.^17^ Higher anxiety levels are associated with alcohol and drug use as coping mechanisms, impairment of bodily functions, stress, depression, and suicidal ideation.^4^,^17^ Nearly half of the nurses in the Labrague and De Los Santos study were unsure if they were prepared to care for COVID-19 patients, resulting in increased anxiety levels.^17^ Of these nurses, over half were unsure or unwilling to care for COVID-19 patients.^17^

This study noted that female nurses were more likely to be distressed compared with male nurses and two times more likely to experience a greater psychological burden.^18^,^19^ These findings are consistent with previous reports.^18^,^19^ Limited studies have been conducted on gender-based anxiety; however, this isn't currently well understood.^20^,^21^ Luxton, Skopp, and Maguen conducted a study on depression and PTSD after combat deployment and reported that female soldiers had higher rates of PTSD than male soldiers.^21^ Although the study participants weren't nurses, the results may be applicable because both groups work in high-intensity environments and crisis situations.

A possible explanation for the disparity in depression and anxiety rates is that historically women report more honest responses to questions regarding anxiety.^21^ The ability to recall events, interpret, and self-report varies between men and women.^22^ Mo and colleagues also reported that female nurses who were only children had higher stress loads due to the internal conflict of working in the COVID-19 environment and being the only social support for older family members.^5^ Nurses with children or those who cared for older family members also had higher anxiety levels.^23^

An unexpected finding in this study was that nurses who didn't provide direct patient care for suspected or confirmed COVID-19 patients had statistically significant higher reported depression and anxiety scores. Literature is scant on the various nursing roles, scope of practice, and prevalence rates of depression and anxiety during a pandemic. This study found that CAs/SNs and LVNs reported higher levels of depression and anxiety than RNs or APNs. These results are consistent with previous studies that also reported an association between nursing practice roles and increased depression and anxiety.^24^,^25^ However, these studies weren't exclusive to the nursing occupation. In contrast, Pouralizadeh and colleagues reported no statistically significant correlation between nurses' practice roles and anxiety.^26^

Potential causes of increased depression and anxiety in CAs/SNs and LVNs may be attributed to their scope of practice and exposure to COVID-19 patients. Limited access to educational resources and inadequate support and oversight by RNs and APNs due to the stressful environment and working conditions may also be contributing factors.

## Limitations

This study had several limitations. Causality can't be established due to the cross-sectional nature of the study. The voluntary web-based survey may have created a selection bias. There were more female participants than male participants, which may have caused a self-selection bias. The results may not be generalizable due to the study taking place at only one organization.

Future research should focus on gathering a larger sample size of nurse participants, recruiting more male nurses, and participating in a multicenter study to validate reported findings. In addition, future studies should aim to investigate factors that correlate nursing roles and scope of practice with depression and anxiety prevalence. Additionally, factors that affect and improve nurses' resilience to reduce depression and anxiety should be investigated.

## Implications for nurse leaders

The COVID-19 pandemic has highlighted a gap in the mental health needs of nurses during extended high-intensity conditions. Nurse leaders play an important role in reducing adversity and stress in nurses by creating an environment that nurtures personal resilience and social and organizational support.^17^,^19^ An emphasis for nurse leaders is to develop intervention programs to mitigate adverse mental health consequences.^17^ Positive outcomes are associated with early identification and organizational intervention.^4^,^17^ Although few evidence-based practice interventions exist at this time, several strategies to implement during epidemic/pandemic situations have been identified.^3^,^8^,^27^,^28^

These strategies can be divided into professional and personal environment needs. Professional environment needs include clear communication regarding updates on the infectious disease, use and management of PPE, patient flow, and policy changes. Providing easily accessible training and care protocols may reduce anxiety associated with unfamiliarity and feelings of lack of control.^3^,^29^ Information may be provided through daily huddles, concise and easy-to-read written communication, or using audiovisual tools. Personal environment needs include scheduled availability of mental health and spiritual professionals during both work and off-work hours. Consider providing an area dedicated to meditation or relaxation and implementing a 30-second “stretch and flex” session every 4 hours.^29^ The physical therapy department can assist in designing a short, realistic workout specific to staff needs. Also encourage staff members to vent their feelings through verbal or written methods.^29^

Preplanning and implementation of mental health action plans are essential to enhance psychological resilience and strengthen the healthcare system and nursing workforce.^4^,^30^,^31^ Early recognition of symptoms and organizational and social support can improve personal resilience and reduce compassion fatigue, burnout, depression, and anxiety.^2^,^32^,^33^

## Support and strengthen

This study noted that depression and anxiety were prevalent among all levels of nursing staff, although CAs, SNs, and LVNs were particularly vulnerable to variable negative mental health outcomes. Moreover, elevated stress levels were associated with clinically significant depression and anxiety. If left untreated, psychological distress can have long-term negative consequences that lead to burnout and poor patient care. Therefore, it's imperative that hospital systems develop supportive plans for nurses that are tailored to strengthen their mental health as part of disaster preparedness.
